# An Introduction to Murine Models of Atrial Fibrillation

**DOI:** 10.3389/fphys.2012.00296

**Published:** 2012-08-03

**Authors:** Genna Riley, Fahima Syeda, Paulus Kirchhof, Larissa Fabritz

**Affiliations:** ^1^Centre for Cardiovascular Sciences, School of Clinical and Experimental Medicine, University of BirminghamBirmingham, UK; ^2^Department of Cardiology and Angiology, University Hospital MünsterMünster, Germany

**Keywords:** atrial fibrillation, atrial tachycardia, cardiac function, electrocardiogram, electrophysiology, mouse heart, murine model, sick sinus syndrome

## Abstract

Understanding the mechanism of re-entrant arrhythmias in the past 30 years has allowed the development of almost curative therapies for many rhythm disturbances. The complex, polymorphic arrhythmias of atrial fibrillation (AF) and sudden death are, unfortunately, not yet well understood, and hence still in need of adequate therapy. AF contributes markedly to morbidity and mortality in aging Western populations. In the past decade, many genetically altered murine models have been described and characterized. Here, we review genetically altered murine models of AF; powerful tools that will enable a better understanding of the mechanisms of AF and the assessment of novel therapeutic interventions.

## Introduction

Atrial fibrillation (AF) affects 1–2% of the population in Europe, and this number is expected to increase twofold to threefold in the next decade, due to both an age-dependent increase in AF and an increased incidence of the arrhythmia (Camm et al., 2010). Even when treated according to best current knowledge, AF remains associated with high residual morbidity and an excess mortality (Camm et al., 2010) that calls for better understanding, diagnosis, and therapy of the arrhythmia rather than the management of symptoms alone. The considered use of animal models has facilitated the characterization of at least four positive feedback loops that contribute to the development of AF (Schotten et al., 2011). Two of these loops augment Ca^2+^ loading and alter ion channel dynamics, which result in the shortening of atrial action potential duration (APD) and focal ectopic activity. Both can be treated with anti-arrhythmic drugs and catheter ablation (Kirchhof et al., 2009) and these techniques can help prevent AF recurrence in selected patients, but the overall recurrence rate of AF remains high.

Unfortunately, some patients with AF are predisposed to recurrent arrhythmias even on such therapy. The changes that predispose the atria toward recurrence of AF have been broadly summarized as “structural remodeling”. Better characterization of these processes are required to enable the identification of patients at risk of recurrence and to develop new therapies for the arrhythmia (Schotten et al., 2011; Wakili et al., 2011).

### Of mice and men

There is no doubt that the best model in which to study human disease mechanisms is the patient. However, many of the molecular changes that confer AF are more pronounced in left atrial tissue (Kahr et al., 2011), which is not readily accessible in patients. Furthermore, the polygenic and multi-factorial processes that govern AF render genetically modified models attractive tools in which to dissect the molecular mechanisms of arrhythmia. Models facilitate the control of confounding factors, enable demonstration of causation rather than association and offer opportunities to validate new therapies in an experimental setting. In recent years, owing to its small size, short gestation period, rapid maturation, and the relative ease with which genetic alterations can be achieved, the mouse has become an attractive mammalian model in which to investigate a number of human conditions. Inbred murine models reduce genetic and environmental variability, enabling disease progression and the effect of genetic modifiers including gender, diet, and physical activity on pathology, to be studied in a closed, complex physiological system. Historically, cardiac arrhythmias were studied in larger mammals such as the goat, pig, or dog, as it was believed that arrhythmia did not occur in mice due to their lack of critical cardiac mass (Janse and Rosen, 2006). Furthermore, the hearts of larger mammals such as non-human primates and dogs are more akin to the human heart than rabbits or smaller rodent species (Russell and Proctor, 2006). However, the seminal and relatively recent demonstrations, disproving the theory of critical cardiac mass (Vaidya et al., 1999), have promoted the use of mice in the study of cardiac arrhythmias. There is considerable genetic homology between humans and mice and this is reflected in the conservation of cardiac developmental pathways, morphological structure, and signaling pathways. There are however, inevitable differences between the murine and human heart: the murine heart rate is up to 10 times faster than that of the human heart and the murine APD is shorter and lacks the typical plateau phase (Fabritz et al., 2003). Yet the relationship between electrical diastole and APD in the mouse is comparable with that of humans. In summary, whilst the mouse provides an excellent model system for primary investigation, molecular underpinnings, and proof of principle strategies, all findings need to be confirmed in other animal models and *in vitro* systems prior to considering clinical application.

### Murine models of atrial fibrillation

**Figure 1:**
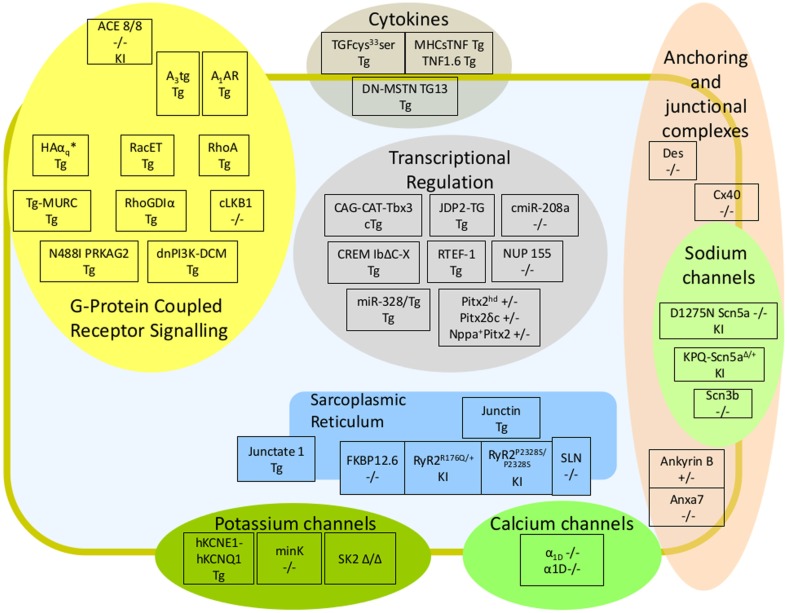
**Murine Models of Atrial Fibrillation**. Schematic diagram depicting murine models of atrial arrhythmia within the context of arrhythmic signaling pathway and subcellular localization. Models were subdivided into altered G-Protein Coupled Receptor signaling; altered ion channels dynamics, anchoring, and junctional complexes; altered calcium homeostasis; altered transcriptional, post transcriptional, and epigenetic regulators; and cytokines and growth factors. The genotype of knockout models associated with atrial fibrillation is provided; knockin models are followed by the suffix (KI), and transgenic models by the suffix (Tg). A brief description of each model, the experimental circumstances used to elicit AF and references can be found within Table 1.

In terms of cardiac assessment, the pipelines of large-scale phenotyping consortia are usually limited to the analysis of blood chemistry, the assessment of heart weight relative to tibial length and the assessment of left ventricular function which, whilst good indicators of cardiac dysfunction, will no doubt often fail to identify an arrhythmic phenotype. Electrocardiograms (ECGs) in lightly sedated and freely roaming mice can be used to diagnose atrial arrhythmias. To study electrophysiological mechanisms of atrial arrhythmias, the use of isolated, beating, perfused hearts have been invaluable. These can be subjected to catheter-based recording of electrograms and action potentials; to electrical stimulation and other arrhythmia provocation techniques, and can be used in conjunction with optical mapping to visualize membrane potential and calcium transients (Eloff et al., 2001; Mathur et al., 2009). These techniques, first developed to study ventricular arrhythmias in mice, have more recently been adapted to study atrial arrhythmias as well (de Diego et al., 2008; Lang et al., 2011). There has been a recent increase in the number of murine models reported to harbor atrial arrhythmias, some of which have been reviewed before (Schotten et al., 2011; Wakili et al., 2011). In this review, murine models were grouped according to the molecular signaling pathway with which they have been most associated as follows: alterations in: G-protein coupled receptor (GPCR) signaling; ion channel dynamics, anchoring, and junctional complexes, calcium homeostasis; transcriptional, post transcriptional, and epigenetic regulation; cytokines and growth factors. In this review we have provided a simplified schematic depicting the position of these murine models both within the context of previously established molecular signaling pathways of atrial arrhythmia and subcellular compartment (Figure 1). We have also generated a tabulated list of murine models in which AF or a surrogate parameter was reported (Table 1). The table indicates whether structural atrial changes were found and gives an idea of whether AF occurred spontaneously under free roaming conditions, under anesthesia, or was provoked during programmed stimulation.

**Table 1:** **Murine Models of Atrial Fibrillation**.

Mouse mutant	Description of transgenic (promoter)	Increased AT/AF telemetric ECG	Increased AT/AF in sedated mice	Increased AT/AF in EP study	AVB	Conduction	A APD	A ERP	Contractile function	Atrial dilation	Atrial thrombi	Atrial fibrosis	Reference
**ALTERED G-PROTEIN COUPLED RECEPTOR SIGNALING**													
ACE 8/8^−/−^ (KI)	Knockin mutant of α*Mhc* promoter at angiotensin I converting enzyme locus (α*Mhc*)	√			√				V=	√		√	Xiao et al. (2004)
A_1_AR (Tg)	Transgenic mutant overexpressing adenosine A1 receptor (α*Mhc*)			√	√		=		**=**	×			Matherne et al. (1997), Kirchhof et al. (2003)
A_3_tg (Tg)	Transgenic mutant overexpressing adenosine A3 receptor (α*Mhc*)			√	√				↓	√		√	Black et al. (2002), Fabritz et al. (2004)
HAα_q_*(Tg)	Transgenic mutant overexpressing constitutively active Gαq (α*Mhc*)	√	√	√	√	√	↑		↓	√	√	√	Mende et al. (1998), Hirose et al. (2009), Laakmann et al. (2010)
RhoA (Tg)	Transgenic mutant overexpressing constitutively active Rho A (α*Mhc*)		√		√				↓	√		√	Sah et al. (1999)
RhoGDIα (Tg)	Transgenic mutant overexpressing bovine Rho GDP dissociation inhibitor α (α*Mhc*)		√		√			↑	**V=**	√		×	Wei et al. (2004)
RacET (Tg)	Transgenic mutant overexpressing constitutively active Rac1 (α*Mhc*)		√	√	√	√	=	=	↓	√		√	Sussman et al. (2000), Adam et al. (2007), Reil et al. (2010)
cLKB1^−/−^	Conditional knockout of serine/threonine kinase 11 using α*Mhc-cre*.		√						↓	√	√	√	Bardeesy et al. (2002), Ikeda et al. (2009)
Tg-MURC (Tg)	Transgenic mutant overexpressing muscle-related coiled-coil protein (α*Mhc*)		√		√				↓	√	√	√	Ogata et al. (2008)
N488I PRKAG2 (Tg)	Transgenic mutant overexpressing human protein kinase AMP-activated gamma 2 subunit carrying N488I mutation (α*Mhc*)	√			×	×			V↓	√			Arad et al. (2003)
dnPI3K-DCM (Tg)	Double transgenic mutant overexpressing dominant negative phosphatidyl inositol 3 kinase lacking kinase activity and overexpression of macrophage stimulating 1 (both α*Mhc*)	√	√		√				↓	√	√	√	Pretorius et al. (2009)
**ALTERED ION CHANNEL DYNAMICS, ANCHORING, AND JUNCTIONAL COMPLEXES AND CALCIUM HOMEOSTASIS**													
**Potassium channels**													
minK^−/−^	Systemic knockout of the KCNE1 (minK) potassium channel	√		√		√	↓		=				Kupershmidt et al. (1999), Temple et al. (2005)
SK2 ^Δ/Δ^	Systemic knockout of the SK2 potassium channel			√	√		↑	=	**=**				Bond et al. (2004), Li et al. (2009)
hKCNE1-hKCNQ1 (Tg)	Transgenic mutant overexpressing hKCNE1-hKCNQ1 fusion protein (α*Mhc*)			√		=	↓	=	**=**				Marx et al. (2002), Chiello Tracy et al. (2003), Sampson et al. (2008)
**Sodium channels**													
Scn5a^Δ/+^ (KI)	Knockin mutant expressing Scn5a sodium channel subunit with 1505-KPQ-1507 deletion			√	√	√	↑	↑	=	√	×	×	Nuyens et al. (2001), Dautova et al. (2009), Guzadhur et al. (2010), Blana et al. (2010)
Scn3b^−/−^	Systemic knockout of Scn3b sodium channel subunit			√	√		↓						Hakim et al. (2008), Hakim et al. (2010)
D1275N Scn5a^−/−^ (KI)	Knockin mutant expressing Scn5a sodium channel subunit with D1275N mutation		√		√	√	↑		V↓				Watanabe et al. (2011)
**Anchoring and junctional complexes**													
Des^−/−^	Systemic knockout of desmin			√	=	√		↓				√	Li et al. (1996), Milner et al. (1996), Schrickel et al. (2010)
Cx40^−/−^	Systemic knockout of connexin40			√	√	↓		↑					Kirchhoff et al. (1998), Hagendorff et al. (1999), Verheule (1999)
Ankyrin_B_±	Systemic knockout of ankyrin2			√			↓						Scotland et al. (1998), Cunha et al. (2011)
**Altered calcium homeostasis**													
α1D^−/−^	Systemic knockout of the α1D subunit of the L-type Ca^2+^ channel		√	√	√	↓	=			×			Platzer et al. (2000), Mancarella et al. (2008)
α_1D_^−/−^	Systemic knockout of the α1D subunit of the L-type Ca^2+^ channel			√	√		↑	=					Namkung et al. (2001), Zhang et al. (2002a), Zhang et al. (2005)
SLN^−/−^	Systemic knockout of sarcolipin		√				↑		A/V↑			√	Babu et al. (2007a), Xie et al. (2012)
RyR2^R176Q/+^ (KI)	Knockin mutant expressing ryanodine receptor 2 carrying R176Q mutation			√		=	=	=		×		×	Chelu et al. (2009)
RyR2^P2328S/P2328S^ (KI)	Knockin mutant expressing Ryanodine Receptor 2 carrying P2328S mutation			√		=	=	=					Goddard et al. (2008), Zhang et al. (2011)
FKBP12.6^−/−^	Systemic knockout of FK506 binding protein 12.6			√		=		=		×		×	Wehrens et al. (2003), Sood et al. (2008), Li et al. (2012)
Junctate 1 (Tg)	Transgenic mutant overexpressing Junctate 1 (α*Mhc*)		√						↓	√	√	√	Hong et al. (2008)
Junctin (Tg)	Transgenic mutant overexpressing canine junctin (α*Mhc*)		√				↑		↓	√	√	√	Hong et al. (2002), Kirchhefer et al. (2004)
Anxa7^−/−^	Systemic knockout of annexin Aa7			√	√			=	**=**				Herr et al. (2001), Schrickel et al. (2007)
**ALTERED TRANSCRIPTIONAL, POST TRANSCRIPTIONAL, AND EPIGENETIC REGULATORS**													
CREM-IbΔC-X (Tg)	Transgenic mutant expressing human cardiac CREM isoform (α*Mhc*)	√	√	√	√		↑		V ↑	√	√		Müller et al. (2005), Kirchhof et al. (2011a)									A ↓	
miR-328/Tg (Tg)	Transgenic mutant overexpressing premiR-328 (α*Mhc*)		√				↓				√		Lu et al. (2010)
cmiR-208a^−/−^	Conditional knockout of miR-208a using β*Actn-cre*		√						↓				Callis et al. (2009)
Pitx2 δc^+/−^	Systemic knockout of Pitx2c isoform			√	**=**		↓		=	×			Liu et al. (2002), Kirchhof et al. (2011b)
Pitx2^hd+/−^	Systemic knockout of Pitx2			√									Lu et al. (1999), Wang et al. (2010)
Nppa^+^ Pitx2^−/−^	Conditional knockout of Pitx2 using *Nppa-cre*									√			Gage et al. (1999), Chinchilla et al. (2011)
cTg-CAG-CAT-Tbx3 (Tg)	Conditional transgenic overexpressing human T-box 3 crossed to *Nppa-cre*		√		√							√	Hoogaars et al. (2007)
NUP155^+/−^	Systemic knockout of nucleoporin 155	√								×			Zhang et al. (2008)
JDP-Tg (Tet-JDP2/α-MHC-tTA)^+/−^	Tetracycline-regulated overexpression of Jun dimerization protein 2 using α*Mhc-tTA*		√		√	↓			=	√		×	Kehat et al. (2006)
RTEF-1 (Tg)	Transgenic mutant overexpressing human TEA domain family member 4 (α*Mhc*)	√	√	√	√	↓				√	√		Chen et al. (2004)
**Cytokines/Growth Factors**													
MHC-TGFcys^33^ser (Tg)	Transgenic mutant overexpressing human TGF-beta 1 carrying C33S mutation (α*Mhc*)			√	√		=	=		×		√	Nakajima et al. (2000), Verheule et al. (2004), Choi et al. (2012)
MHCsTNF (Tg)	Transgenic mutant overexpressing tumor necrosis factor (α*Mhc*)		√	√	√			↑		√			Li et al. (2000), Sivasubramanian et al. (2001), Sawaya et al. (2007)
TNF1.6 (Tg)	Transgenic mutant overexpressing tumor necrosis factor (α*Mhc*)	√		√	√		=		A	√	√	√	Kubota et al. (1997), Saba et al. (2005)									↓	
DN-MSTN TG13 (Tg)	Transgenic mutant overexpressing inhibitory N-terminal pro-peptide (α*Mhc*)		√	√	√				**=/**↑	√		√	Rosenberg et al. (2012)

*Murine models of AF were divided into five categories: Altered G-Protein Coupled signaling; altered ion channel dynamics, anchoring, and junctional complexes, altered calcium homeostasis; altered transcriptional, post transcriptional, and epigenetic regulators; cytokines and growth factors. Models were identified via a publication search on Pubmed using the terms “mouse atrial arrhythmia.” This returned a total of 351 hits all which were then individually screened for relevance in terms of: spontaneous atrial tachycardia and/or fibrillation (AT/AF) during telemetric or sedated analysis; increased induced occurrence of atrial tachycardia and/or fibrillation (AT/AF) in electrophysiology (EP) studies; atrial ventricular block (AVB) including PQ/PR prolongation as AVBI; effect on atrial conduction (direct and indirect); effect on atrial action potential duration (APD); effect on atrial effective refractory period (ERP); effect on contractile function refractory period, ERP; contractile function (V-ventricular, A-atrial); presence of atrial dilation; atrial thrombi, and atrial fibrosis. √ indicates presence of that condition, × indicates absence of the condition, unticked boxes do not necessarily mean that a condition has been completely ruled out for a given model, only that there is no evidence as *yet*. =, unaltered. References are shown in the right hand column. The first reference for each model describes the generation of that model*.

In the following text we will highlight several of these models to exemplify the complex and divergent changes that can result in murine AF. We hope that understanding mechanisms of arrhythmia in such models will further our understanding and treatment of AF in humans.

## Models of AF with Altered G-Protein Coupled Receptor Signaling

Systemically, GPCRs are involved in extracellular-intracellular signaling and underpin a wide variety of biological responses (Neer, 1994; Park et al., 2004). The most commonly studied GPCRs in the heart include the adrenergic, angiotensin, endothelin, and adenosine receptors (Salazar et al., 2007).

### Angiotensin II

The role of renin-angiotensin signaling within cardiac pathological states, particularly in regard to blood pressure, is well documented. However, there is increasing evidence that angiotensin II exerts a more local stimulatory effect on heart function (Sancho et al., 1976; Lindpaintner et al., 1988; De Mello and Frohlich, 2011). Furthermore, atrial angiotensin II expression levels are increased in patients with AF (Boldt et al., 2003; Cong et al., 2010; De Jong et al., 2011). *In vitro*, rapid pacing of atrial myocytes increased the paracrine secretion of angiotensin II (Tsai et al., 2011), suggesting that fibrillation promotes angiotensin II production and therefore signaling. Cardiac-specific overexpression of angiotensin I converting enzyme (ACE), a peptidase that converts angiotensin I to its biologically active counterpart, angiotensin II, in mice, resulted in atrial dilation, fibrosis, and spontaneous AF under free roaming conditions (Xiao et al., 2004), suggesting that increased angiotensin II signaling promotes the development of AF. Yet, cardiac-specific overexpression of the angiotensin receptor AT_1a_ caused marked atrial enlargement, bradycardia, and abnormal atrio-ventricular (AV) conduction, but failed to increase AF susceptibility in neonatal mice (Hein et al., 1997).

### Adenosine receptors

Adenosine receptors are a type of purinergic G-Protein coupled receptor, activated by adenosine, that have an inhibitory effect on adenlyl cyclase signaling (and therefore cAMP levels) and voltage-gated ion channels, whilst concomitantly activating potassium channels (Priori et al., 1993; Headrick et al., 2011). Short-term adenosine A1 receptor (A_1_AR) stimulation induces bradycardia and AV block in humans (DiMarco et al., 1983). Transgenic models of adenosine A1 and A3 receptors were developed primarily to evaluate the protective effect of receptor overexpression on myocardial ischemia (Matherne et al., 1997). However, enhanced expression of either A1 or A3 adenosine receptors also provoked atrial bradycardia and AV block and increased susceptibility to AF dependent on the degree of bradycardia (“tachycardia-bradycardia syndrome”; Kirchhof et al., 2003; Fabritz et al., 2004), that could potentially lead to bradycardiomyopathy (Fabritz et al., 2004). Therefore, increased cAMP levels (as shown by this model and that of ACE overexpression) appear to contribute to the development of AF.

### Galpha Q

Heterotrimeric G-proteins are membrane-associated complexes that comprise an alpha, beta, and gamma subunit and are the intracellular effectors of GPCRs (Neer, 1994; Park et al., 2004; Salazar et al., 2007). In the heart, Gαq associates with alpha1-adrenergic, endothelin (ET_A_), and angiotensin II type I (AT_1_) receptors. Following ligand activation, Gαq is phosphorylated which results in the activation of numerous downstream effectors including phospholipase C (PLC), protein kinase C (PKC), thymoma viral protein-oncogene (Akt), and Ras homologous (Rho; Salazar et al., 2007; Filtz et al., 2009; Ben-Ami et al., 2011; Pfreimer et al., 2012). To investigate the putative role of Gαq in cardiac hypertrophy, two transgenic mouse lines overexpressing endogenous and a constitutively active *G*α*q* subunit under the control of the α*Mhc* promoter were independently generated (D’Angelo et al., 1997; Mende et al., 1998). Both models developed cardiac hypertrophy in the absence of myocyte disarray or necrosis, followed by diffuse atrial and ventricular fibrosis and heart failure (D’Angelo et al., 1997; Mende et al., 1998; Hirose et al., 2009). A number of these mice developed left atrial thrombi, another pathological characteristic associated with AF (Mende et al., 1998; Laakmann et al., 2010), and indeed, AF was recorded as occurring spontaneously under roaming conditions (Hirose et al., 2009). Similar to the Ras homolog gene family member A (*RhoA*) overexpression model discussed below, there is recent evidence to suggest that in this model, AF may be a primary pathology within its own right that does not occur secondary to ventricular remodeling (Sah et al., 1999; Laakmann et al., 2010).

### Ras homolog gene family member A

Initially identified for its role within hypertrophic signaling pathways (Sah et al., 1996), RHOA is a small membrane-associated GTPase involved in actin cytoskeleton organization (Ridley and Hall, 1992). Mice overexpressing either wild type RHOA or an activated form of RHOA under the control of the cardiac-specific α*Mhc* promoter died prematurely and developed cardiac enlargement, cellular hypertrophy, interstitial fibrosis, and heart failure (Sah et al., 1999). Atrial enlargement was more pronounced than ventricular enlargement, suggesting atrial dilation preceded ventricular dilation, which is consistent with the reported temporal cardiac expression profile of α*Mhc* (Chizzonite et al., 1982; Sweeney et al., 1985; Bouvagnet et al., 1987; Colbert et al., 1997). Electrocardiography (ECG) performed under anesthesia was indicative of AF and AV block (Sah et al., 1999).

More recently overexpression of RhoGDIα, an endogenous specific GDP dissociation inhibitor for all Rho family proteins, led to atrial arrhythmias and mild ventricular hypertrophy (Wei et al., 2004). ECG and intracardiac electrophysiological analysis showed these mice developed bradycardia, AV block, and atrial arrhythmias concomitant with reduced expression of the gap junction protein, connexin 40, before the onset of cardiac hypertrophy and heart failure (Wei et al., 2004). Taken together these murine models suggest that altered expression levels of Rho family proteins can propagate a pro-arrhythmic environment.

### Rac1

Rat sarcoma (RAS)-related C3 botulinum toxin substrate 1 (Rac1) has long been recognized for its role in actin cytoskeleton organization, and is a plasma membrane-associated GTPase belonging to the RAS superfamily (Ridley and Hall, 1992; Sussman et al., 2000). Homozygous mice overexpressing constitutively active *Rac1* under the control of the α*Mhc* promoter were found to be viable (Chizzonite et al., 1982). However, a proportion of progeny died within 2 weeks of birth owing to increased postnatal α*Mhc* promoter activity as a result of increased circulating levels of thyroid hormone (Chizzonite et al., 1982; Sussman et al., 2000). Mice surviving this initial vulnerable period achieved survival comparable with that of wild type (Sussman et al., 2000), but by 2 months of age, mutants developed atrial enlargement and atrial wall thinning juxtaposed to ventricular wall thickening and reduced ventricular chamber size (Sussman et al., 2000). Interstitial fibrosis and systolic heart failure progressively developed and 75% of the transgenic mice developed spontaneous AF under anesthesia (Adam et al., 2007).

### Other models of altered GPCR signaling

Several other murine models of AF have been generated by the genetic manipulation of other proteins within GPCR signaling cascades using α*Mhc* promoter-driven expression. Briefly, models reported to develop spontaneous AF under anesthesia include a knockout of serine/threonine kinase 11 (Stk11/Lkb1; Bardeesy et al., 2002; Ikeda et al., 2009; Pretorius et al., 2009); a transgenic mouse overexpressing Muscle-Related Coiled-Coil protein (MURC1; Ogata et al., 2008) and a transgenic mouse overexpressing human protein kinase AMP-activated gamma 2 subunit (PRKAG2) carrying the N488I mutation (Arad et al., 2003). A double transgenic mutant overexpressing dominant negative phosphatidyl inositol 3 kinase and macrophage stimulating 1 (Mst1) was also reported to develop spontaneous AF during telemetry (Pretorius et al., 2009).

## Models of AF with Altered Ion Channel Dynamics, Anchoring, and Junctional Complexes and Calcium Homeostasis

### Ion channel dynamics

#### Potassium channels

Potassium ion channels remove K^+^ from the cell defining the repolarization phase of the action potential. Therefore, any genetic modification that alters the expression levels or gating of potassium channels has the potential to alter the cardiac action potential and thereby act as an arrhythmic substrate. Models include: the systemic knockout of the small conductance calcium-activated potassium channel (SK2) which increased susceptibility to AF induction (Bond et al., 2004; Li et al., 2009), and the systemic knockout of the voltage-gated subfamily E member 1 potassium channel (KCNE1) which resulted in spontaneous AF under telemetry (Kupershmidt et al., 1999; Temple et al., 2005).

#### Sodium channels

##### SCN5A

Sodium currents are responsible for the rapid depolarization of myocytes and are important for conduction, repolarization, and refractoriness. Similar to potassium channel disruption, several animal models in which sodium channel dynamics have been altered develop AF. *SCN5A* encodes the alpha subunit of the cardiac voltage-gated sodium channel (Nav1.5; Gellens et al., 1992). Mutations within *SCN5A* underlie a number of clinically defined arrhythmias including long QT syndrome type 3 (LQT3), Brugada syndrome, progressive conduction disease, and AF (Ruan et al., 2010; Wilde and Brugada, 2011) and many of these diseases harbor a propensity for atrial arrhythmias (Eckardt et al., 2001; Zellerhoff et al., 2009).

Systemic targeted disruption of *Scn5a* results in homozygous lethality whilst heterozygotes display a 50% reduction in sodium conductance (Papadatos et al., 2002). A knockin murine model, Scn5a Δ1505-1507 KPQ (ΔKPQ-Scn5a), of a human mutation in which three amino acids within the inactivation domain of Nav1.5 were deleted, resulted in a persistent inward Na^+^ current thus delaying the repolarization of the action potential and causing longer QT intervals (Wang et al., 1996; Nuyens et al., 2001; Head et al., 2005). ECG recorded by telemetry and under anesthesia revealed that heterozygous mice mimic the long QT syndrome 3 phenotype found in patients (Nuyens et al., 2001). More recently, atrial electrophysiology was assessed in ΔKPQ-Scn5a mutant mice. Concurrent with ventricular action potentials, atrial action potentials were also prolonged (Dautova et al., 2009; Blana et al., 2010). ΔKPQ-Scn5a mice were more susceptible to atrial arrhythmias induced by maneuvers known to provoke torsades de pointes, namely short-long-short stimulation sequences (Blana et al., 2010). Aged ΔKPQ-Scn5a mice were reported to be more susceptible to atrial arrhythmias induced by programmed stimulation (Guzadhur et al., 2010), and have altered sinoatrial node function and intra-atrial conduction (Head et al., 2005; Wu et al., 2012).

Analysis of another knockin murine model, homozygous for the human D1275N mutation (Groenewegen et al., 2003; Remme et al., 2006), reported slowed conduction, atrial arrhythmias, sinus node dysfunction, and progressive AV block, that culminated in sudden death at around 12 weeks of age (Watanabe et al., 2011). Mutations within other accessory subunits of the cardiac sodium channel also affect channel dynamics; homozygous deletion of the beta subunit, *SCN3B*, led to conduction disturbances, bradycardia, and an increased susceptibility to induced atrial arrhythmias (Hakim et al., 2008, 2010; Olesen et al., 2011).

### Anchoring and junctional complexes

#### Connexins

The cardiac voltage sodium channel, Nav1.5, was shown to colocalize with Connexin 43 within the intercalated disk of ventricular cardiomyocytes (Maier et al., 2002). The gap junction proteins Connexin 40 (Cx40, GJA5) and Connexin 43 (Cx43, GJA1) function to electrically couple cardiomyocytes, facilitating syncytial contraction. Cx40 and Cx43 are expressed at equal levels in atria however, only Cx43 is expressed in the ventricles (Lin et al., 2010). Both mutations in *CX40* and *CX43* have been reported in patients with idiopathic AF (Gollob et al., 2006; Thibodeau et al., 2010). Cardiac-specific loss of Cx43 and overexpression of a dominant negative mutant of Cx43 led to the development of spontaneous ventricular arrhythmias (Hong et al., 2008; Sood et al., 2008; Li et al., 2012), consistent with a predominating ventricular role in the mouse. However, systemic loss of Cx40 (or GJA5) in the mouse resulted in an increased propensity to induced atrial arrhythmias and reduced conduction velocity (Kirchhoff et al., 1998; Hagendorff et al., 1999; Verheule, 1999). Furthermore, loss/redistribution of Cx40 appears to be a common feature of other murine models of AF including overexpression of RHOA, overexpression of constitutively active RAC1, cardiac-specific overexpression of ACE, overexpression of Tumor necrosis factor (TNF), and cardiac overexpression of cAMP Response element modulator (CREM; Sah et al., 1999; Kasi et al., 2007; Sawaya et al., 2007; Adam et al., 2010; Kirchhof et al., 2011a; see Table 1 and Figure 1 for further details).

Recent evidence suggests that in the heart of higher vertebrates, the protein complexes that traditionally comprise the cell–cell junctions of the adherens junctions and desmosomes of epithelial cells, actually coordinate to form a single heterogeneous junction referred to as the area composita (Borrmann et al., 2006; Franke et al., 2006). Loss of desmin, an intermediate filament that selectively associates with desmosomal complexes, has been reported to lead to atrial fibrosis and increased susceptibility to AF induction (Li et al., 1996; Milner et al., 1996; Schrickel et al., 2010). Hence, disrupted cell–cell mechanical coupling may also act as a substrate for AF.

#### Ankyrin B

The ankyrin family are membrane-associated adaptor proteins that serve to link membrane-associated proteins with the cytoskeleton (Bennett and Gilligan, 1993; Li et al., 1993). Loss of function of Ankyrin B (ANK2) is associated with long QT syndrome and sudden cardiac death in humans and mice (Mohler et al., 2003). Previously, ankyrin B was shown to regulate cardiac sodium channels dynamics (Chauhan et al., 2000), Na/K ATPase, Na/Ca exchanger 1, and inositol triphosphate (InsP3; Mohler et al., 2005). Loss of ankyrin B was reported to increase cardiac Na^+^ channel opening times, increasing APD (Chauhan et al., 2000). Recent evidence suggests that disrupted ankyrin B function is a predisposing factor for the development of atrial arrhythmias in humans (Cunha et al., 2011). Mice heterozygous for ankyrin B were reported to develop spontaneous atrial arrhythmias under telemetry and ECG studies showed increased susceptibility to AF induction by pacing (Cunha et al., 2011). In the same work, ankyrin B was shown to bind to the alpha 1C subunit of the L-type Ca^2+^ channel (Cav1.2) and loss of ankyrin B resulted in reduced inward Ca^2+^ current and a shortened APD (Cunha et al., 2011). Hence, it would appear that ankyrin B regulates both Na^+^ and Ca^2+^ currents and that disruption of ankyrin B perturbed these currents and lead to spontaneous AF.

### Calcium homeostasis

The voltage-dependent L-type Ca^2+^ channel contributes to the late depolarizing currents of the cardiac action potential. Comprising five subunits in total, the alpha subunit of the L-type Ca^2+^ channel is the transmembrane spanning subunit. In the heart, the alpha 1d subunit (Cav1.3) is exclusively expressed in the atria (Zhang et al., 2005), where it plays an important role in the spontaneous diastolic depolarization of pacemaker cells (Zhang et al., 2002a). Systemic deletion of Cav1.3 rendered mice susceptible to AF induction (Zhang et al., 2005; Mancarella et al., 2008).

Calcium homeostasis is important in the maintenance of normal sinus rhythm and contraction: Ca^2+^ induced Ca^2+^ release is the mainstay of mechanoelectrical coupling, clearance of Ca^2+^ from the cytosol significantly contributes to the late depolarizing currents of the cardiac action potential and Ca^2+^ acts as a secondary messenger within many intracellular signaling pathways. Therefore, any genetic modification that alters the expression levels or gating of calcium channels; or modifies signaling cascades in which Ca^2+^ features as a secondary messenger, has the potential to act as an arrhythmogenic substrate. Previous reviews of AF models have focused on calcium homeostasis therefore, we will only mention them briefly (Schotten et al., 2011; Wakili et al., 2011).

Murine models with mutations in the ryanodine receptor and associated proteins are reported to be susceptible to AF (see Table 1). Models include: mice carrying the human mutation, R176Q, within the outward Ca^2+^ channel of the sarcoplasmic reticulum, ryanodine receptor 2 (RyR2; Chelu et al., 2009); mice carrying the human mutation, P2328S, within RyR2 (Goddard et al., 2008; Zhang et al., 2011); systemic deletion of the RyR2 binding protein, FKBP12.6 (Wehrens et al., 2003; Sood et al., 2008; Li et al., 2012); cardiac-specific overexpression of the Ca^2+^ binding protein, junctate 1 (Hong et al., 2002, 2008); cardiac-specific overexpression of the sarcoplasmic transmembrane protein, junctin (Hong et al., 2002; Kirchhof et al., 2007), and systemic deletion of the Ca^2+^- and GTP-dependent membrane-associated protein, annexin Aa7 (Anxa7; Herr et al., 2001; Schrickel et al., 2007). Of note, spontaneous AF was observed in mice with a 29-fold overexpression of junctin, whereas mice with 10-fold overexpression showed only reduced adaption of heart rate to stress (Hong et al., 2002; Kirchhof et al., 2007).

Sarcolipin (SLN) is a transmembrane protein located within the sarcoplasmic reticulum that like phospholamban, is a key regulator of sarcoplasmic reticulum Ca^2+^ ATPase (SERCA) function (Odermatt et al., 1998; Asahi et al., 2003; Minamisawa et al., 2003; Babu et al., 2007a; Bhupathy et al., 2009). However, unlike phospholamban, SLN activity is mediated by calcium-calmodulin dependent protein kinase II (CaMKII), not protein kinase A (PKA; Bhupathy et al., 2009). At low Ca^2+^ concentrations, SLN inhibits SERCA, reducing its affinity for Ca^2+^ which has the effect of slowing Ca^2+^ uptake into the sarcoplasmic reticulum, reducing contractility (reduced force generation and speed of relaxation; Tupling et al., 2002). Reduced levels of SLN mRNA and protein expression are associated with chronic AF in humans (Uemura et al., 2004; Shanmugam et al., 2011). Aged homozygous SLN knockout mice (Babu et al., 2007b) were reported to develop atrial fibrosis and spontaneous AF under anesthesia (Xie et al., 2012), suggesting that increased SERCA function promotes AF.

## Models of AF with Altered Transcriptional, Post Transcriptional, and Epigenetic Regulation

Dysregulation of GPCR signaling, ion channel dynamics, and calcium homeostasis have long been associated with the development and maintenance of AF however, the net effect of these pathways upon transcription and the contribution of transcriptional, post transcriptional, and epigenetic dysregulation to AF are less well understood. This represents a rapidly expanding area of AF research and it is here, within this category, the majority of the more recently described animal models of AF reside.

### cAMP response element modulator

cAMP Response element modulator belongs to the cAMP response element binding (CREB)/activating transcription factors (ATF) family of transcription factors and is a phosphorylation target of PKA. Cardiac-directed expression of CREM-IbΔC-X, a human cardiac CREM repressor isoform, resulted in both atrial and ventricular enlargement, atrial wall thinning, atrial thrombus formation, disturbed myocyte architecture, and increased basal left ventricular function (Müller et al., 2005). Echocardiography analysis revealed that atrial chamber dilation preceded the development of AF (Kirchhof et al., 2011a). In addition to reduced wall thickness, atrial myocytes were elongated and atrial weight increased, while neither cellular hypertrophy nor fibrosis were found (Kirchhof et al., 2011a). Telemetric ECG indicated that mutants suffered from atrial ectopies, the occurrence of which increased with age until mice entered an almost constant state of atrial tachycardia (Kirchhof et al., 2011a). Reduced phosphorylation of CREM (due to overexpression of CREM-IbΔC-X) was associated with altered Ca^2+^ homeostasis concurrent with spontaneous Ca^2+^ release and reduced gap junction protein expression (CX40; Kirchhof et al., 2011a). CREM transcription factors bind to the cAMP response element found within several promoters (Foulkes et al., 1991). Whilst many of the genetic targets of CREM involved in spermatogenesis have been identified (Kosir et al., 2012), cardiac-specific gene targets remain largely unknown and are subject to current studies.

### PITX2c

The paired-like homeodomain transcription factor 2 (Pitx2) is a downstream target of Nodal within the transforming growth factor beta (TGFβ) signaling pathway and was primarily recognized as having a role in establishing left-right asymmetry during embryonic development (Meno et al., 1998; Ryan et al., 1998). The highest Pitx2 isoform expressed in the murine and human heart is Pitx2c (Kitamura et al., 1999; Schweickert et al., 2000). Systemic loss of Pitx2c in mice lead to embryonic lethality and resulted in a number of phenotypes including abnormal cardiac morphogenesis (Gage et al., 1999). During embryogenesis, Pitx2c was shown to synergistically drive expression of natriuretic peptide A (*Nppa)* in the presence of the homeobox transcription factor, *Nkx2.5* (Ganga et al., 2003) which in turn, suppressed the expression of the pacemaker channel gene, *Hcn4*, and the T-box transcription factor (*Tbx3*), gene, thus delineating contractile atrial myocardium from the sinoatrial node (Hoogaars et al., 2007; Mommersteeg et al., 2007).

Genome-wide association studies (GWAS) identified sequence variation within 4q25 as conferring increased susceptibility to AF (Schott et al., 1995; Gudbjartsson et al., 2007; Kääb et al., 2009). Hence, owing to its genomic location within this candidate region, *PITX2* was considered a prime candidate gene for the development of atrial arrhythmias in man. Indeed, expression analysis confirmed that *PITX2C* was also expressed in adult left atrial tissue, further supporting a biological function beyond that of embryonic left-right patterning (Wang et al., 2010; Kirchhof et al., 2011b). In mice carrying a hypomorphic allele of *Pitx2c*, sedation and pacing revealed an increased tendency of atrial flutter and tachycardia in heterozygous mutants (Wang et al., 2010). Isolated, beating hearts carrying a heterozygous *Pitx2c*-deletion had a reduced APD and were also susceptible to AF (Kirchhof et al., 2011b). Echocardiography suggested there were no apparent morphological defects (Kirchhof et al., 2011b). In contrast, homozygous deletion of *Pitx2c* in the heart, appeared to provoke structural changes later in life as well as changes in ion channel expression (Chinchilla et al., 2011), which could also offer mechanistic insight into the observed arrhythmic phenotype.

Given its role in suppressing “pacemaker” genes and defining the contractile myocardium, it has been proposed that loss of Pitx2c results in a “less left” atrial phenotype promoting ectopic left atrial activity however, how this is achieved in fully differentiated, adult myocardium is unclear at present.

### T-box transcription factor 3

Similar to loss of Pitx2c, mice over expressing Tbx3, another gene implicated in the specification of pacemaker cells, in a cardiac-specific manner, developed atrial ectopic beats (Hoogaars et al., 2007).

### RTEF-1

Transcription enhancer factor-1-related factor (RTEF-1) is a transcriptional target of alpha1-adrenergic signaling (Stewart et al., 1998; Ueyama et al., 2000). Cardiac-specific overexpression of human RTEF-1 in mice led to the development of spontaneous AF under telemetry, an increased tendency to AF induction by burst pacing in younger mice and the development of sustained AF in older (12 months) transgenic founder mice (Chen et al., 2004). Atrial dilation and thrombi were reported in this transgenic model however, there was no evidence of fibrosis. Owing to reduced atrial conduction velocity, the expression levels of phosphorylated compared to dephosphorylated Cx40 were assessed: The levels of phosphorylated Cx40 were found to be reduced concomitant with an increase in dephosphorylated Cx40 suggesting that gap junction complexes were compromised in this model of AF (Chen et al., 2004).

### Jun dimerization protein 2

c-Jun dimerization protein 2 (JDP2) is a transcriptional repressor belonging to the basic leucine zipper (bZIP) family of transcription factors (Aronheim et al., 1997; Jin et al., 2001). Tetracycline-regulated cardiac-specific overexpression of JDP2 resulted in atrial dilatation and atrial myocyte hypertrophy and reduced conduction velocity (Kehat et al., 2006). Furthermore, spontaneous AF under anesthesia was reported in a “small number” of mice (Kehat et al., 2006). Cx40 protein levels were also perturbed in this model (reduced expression level of total Cx40), which was indicative of the inhibitory effect of JDP2 on Cx40 promoter activity (Kehat et al., 2006). Hence loss of Cx40 may also be an attributing factor to the observed conduction defects and the development of AF in this model.

### Nucleoporin 155

Nucleoporin 155 (NUP155) is a major component of the nuclear pore complex within the nuclear envelope that facilitates the transport of DNA and mRNA from the nucleus to the cytoplasm (Zhang et al., 2002b). Systemic heterozygous deletion of NUP155 in mice, reportedly lead to spontaneous AF under roaming conditions (Zhang et al., 2008). More importantly, a single nucleotide polymorphism that evokes a missense mutation within NUP155 has been identified as contributing to AF in patients (Zhang et al., 2008). *In vitro* analysis showed that this mutation impaired nuclear permeability to *Nup155* gene products (Zhang et al., 2008). NUP155 was recently shown to interact with histone deacetylase 4 (HDAC4), hence this arrhythmic phenotype may also be the consequence of altered transcriptional activity (Kehat et al., 2011).

### MicroRNAs

In recent years a number of microRNAs (miRNA), endogenous non-coding ∼22mer RNA molecules that regulate gene expression by gene silencing, have been increasingly implicated in cardiac pathology (Wakili et al., 2011; Wang et al., 2011). This highlights the potential role of post transcriptional regulation alongside other such control mechanisms as transcriptional regulation, translational regulation, and post-translational modification.

#### miR-328

miR-223, miR-328, and miR-664 were found to be upregulated in a canine model of AF (Yue et al., 1997; Lu et al., 2010). The generation of a transgenic mouse overexpressing miR-328 under the control of the α*Mhc* promoter, resulted in the development of spontaneous AF under anesthesia (Lu et al., 2010). Furthermore loss of miR-328 protected against AF induction via pacing following muscarinic acetylcholine receptor stimulation (Lu et al., 2010). The alpha 1c and beta 1 subunits of the L-type Ca^2+^ channel were identified as targets of miR-328, and the expression levels of the L-type Ca^2+^ channel protein subunits encoded by these genes, Cav1.2 and Cavβ1, were also reduced, suggesting that the observed shorter APD in this model is the result of reduced L-type Ca^2+^ currents (Lu et al., 2010).

#### miR-208

Transgenic mice overexpressing miR-208a displayed reduced cardiac function by 3 months of age and went on to develop first and second degree AV block (Callis et al., 2009). Conversely, loss of miR-208a in a murine knockout model had a protective effect on cardiac remodeling following thoracic aortic banding (van Rooij et al., 2007). Despite the seemingly positive effect of reducing miR-208a on cardiac pathology, miR-208a knockout mice also developed spontaneous AF under anesthesia (Callis et al., 2009). The effect of miR-208b on cardiac conduction has yet to be investigated but it differed from miR-208a by only 3 bp and shared identical seed regions (Callis et al., 2009), hence it seems likely that miR-208b regulates the same targets as miR-208a (Callis et al., 2009) and that both feature within arrhythmogenic signaling pathways. miR-208a and miR-208b are encoded within intron 27 of the α*Mhc* and β*Mhc* genes respectively (van Rooij et al., 2007) and regulatory targets of miR-208a include thyroid hormone associated protein 1 and myostatin (see next paragraph; Callis et al., 2009).

## Cytokines and Growth Factors

### Myostatin

Myostatin was among the first proteins to be identified as a negative regulator of hypertrophy (McPherron et al., 1997). Very recently, cardiac-specific expression of the inhibitory N-terminus of myostatin pro-peptide was shown to increase susceptibility to AF induction in mice (Rosenberg et al., 2012). Taken together, these results suggest that loss of myostatin expression can act as a substrate for AF. Preliminary data has shown the voltage-gated potassium channel Kv1.4 (KCNA4) to be upregulated in this model, although the impact of this on APD has yet to be determined (Callis et al., 2009; Rosenberg et al., 2012).

### Transforming growth factor beta 1

Transforming growth factor beta 1 (TGFß1) is a cytokine that regulates numerous cellular processes including growth, differentiation, adhesion, migration, and apoptosis. However, in the adult heart, TGFß1 is a key regulator of fibrosis. Enhanced fibrosis reduces the electrical coupling of cardiomyocytes (Shaw and Rudy, 1997; Allessie et al., 2005). The atria have been shown to be more susceptible to fibrotic remodeling (Burstein et al., 2008) and pacing of cultured atrial-derived myocytes was shown to increase expression of TGFβ1 (Yeh et al., 2011). In patients, increased expression levels of TGFß1 are associated with chronic AF, valvular heart disease, atherosclerosis, and pressure overload (Lamirault et al., 2006; Xu et al., 2010; Creemers and Pinto, 2011). In mice, cardiac-specific overexpression of constitutively active TGFß1 caused atrial fibrosis, atrial conduction disturbances, and AF (Verheule et al., 2004). Recent data suggested that triggered activity arising due to reduced APD and spontaneous Ca^2+^ release, contribute to the development of AF in this murine model (Choi et al., 2012). Increased levels of angiotensin II and reactive oxygen species were shown to increase the promoter activity of *Tgf*β*1*, connective tissue growth factor (*Ctgf*), and collagen in fibroblasts thus perpetuating a profibrotic environment that could promote conduction defects (Hao et al., 2011; Tsai et al., 2011).

### Tumor necrosis factor

Tumor necrosis factor (formerly referred to as TNFα) is another inflammatory cytokine implicated in the development and pathology of AF in humans (Cao et al., 2011; Deng et al., 2011). Cardiac-specific overexpression of TNFα in mice resulted in atrial dilation, fibrosis, thrombi development, and spontaneous AF under roaming conditions (Kubota et al., 1997; Saba et al., 2005). In a second TNF overexpression model, downregulation of the gap junction protein Connexin 40 was also reported (Sawaya et al., 2007).

## Conclusion

A long list of murine models that confer increased susceptibility to atrial arrhythmias, further substantiates the value of genetically altered murine models in advancing our understanding of the processes that initiate and propagate AF. In this review, models were grouped into signaling pathways already associated with AF. However, this is not to say that the proteins they encode are restricted to any single pathway. Each “pathway” most likely represents a small component of a much larger, more complex, pro-arrhythmic pathway.

In the vast majority of models, atrial arrhythmia was reported to be induced in a non-physiological setting such as anesthesia or pacing (more than three quarters of mouse mutants reported in Table 1). The relationship between anesthesia and AF is still of clinical relevance (Laakmann et al., 2010). However pacing induced arrhythmia suggests that whilst there is a substrate for atrial arrhythmia, this can be well tolerated and undetected in the absence of a triggering factor. Reported cases of spontaneous atrial arrhythmias in freely roaming mice were relatively rare (less than a quarter of the mouse mutants reported in Table 1) and almost always accompanied by atrial enlargement and/or fibrosis. We were intrigued to find that the majority of models reported to develop spontaneous AF without anesthesia or interventions were transgenic overexpression mutants. High levels of overexpression may have a toxic effect on the cell that also contributes to atrial phenotype, whereby overexpression leads to structural changes beyond those observed during “normal” pathophysiology. It is also possible that such expression levels may actually trigger a common final pathological pathway that mimics human pathology. Indeed the majority of the mutant models discussed in this review displayed dose dependent phenotypes that arose due to the heterozygous or homozygous expression and/or the positional effect of transgene integration (D’Angelo et al., 1997; Mende et al., 1998; Sussman et al., 2000; Fabritz et al., 2004; Kasi et al., 2007). The differential onset of α*Mhc* promoter expression during embryogenesis in the atria and at birth in the ventricles (Chizzonite et al., 1982; Sweeney et al., 1985; Bouvagnet et al., 1987; Colbert et al., 1997; Hein et al., 1997; Wei et al., 2004) might also mitigate a gene dosage effect across the heart.

Atrial fibrillation is a complex arrhythmia, and is likely to depend on multiple intertwined mechanisms. While the principal dysregulations in ion channel function and intracellular calcium homeostasis have been characterized, more recent genetic findings suggest that dysregulation of gene transcription and an imbalance in major regulatory pathways of cell function may contribute to the complex genesis of AF. Future challenges include the identification and investigation of the downstream components of these pathways and henceforth, the identification of therapeutic targets. The use of GWAS will lead to the development of new models of AF that better mimic human pathology from a different perspective. The differences between man and mice notwithstanding, murine models offer unique opportunities to investigate the functional and biochemical consequences of such complex regulatory changes, and to integrate these domains into our understanding of the genesis of AF.

## Conflict of Interest Statement

The authors declare that the research was conducted in the absence of any commercial or financial relationships that could be construed as a potential conflict of interest.
